# Upbeat-torsional nystagmus due to a small pontine hemorrhage: A case report

**DOI:** 10.1097/MD.0000000000042064

**Published:** 2025-05-23

**Authors:** Mayuka Kimura, Hiroaki Fujita, Hiroki Onuma, Keisuke Suzuki

**Affiliations:** a Department of Neurology, Dokkyo Medical University, Tochigi, Japan.

**Keywords:** brainstem stroke, case report, dizziness, torsional nystagmus, vertigo

## Abstract

**Rationale:**

Characteristic nystagmus often predicts a responsible lesion and aids in the differential diagnosis of dizziness or vertigo. Upbeat-torsional nystagmus usually presents with posterior canal benign paroxysmal positional vertigo, which occurs after a few seconds of latency following a change in head position. However, it can also be observed in patients with central lesions. This case report aims to highlight the importance of neurologically examining characteristic nystagmus in detecting central lesions.

**Patient concerns:**

A 56-year-old woman suddenly developed dizziness. Neurological examinations revealed torsional nystagmus with a mild upbeat component that was not triggered by head position changes. No other neurological manifestations were present.

**Diagnosis:**

Brain magnetic resonance imaging revealed a left dorsal pontine hemorrhage.

**Interventions:**

The patient received antihypertensive treatment. Adenosine triphosphate disodium hydrate was administered as a symptomatic treatment for the patient’s dizziness.

**Outcomes:**

The torsional nystagmus gradually decreased in frequency and resolved around the 4th day of hospitalization. The patient was able to walk steadily and was discharged home on the 14th day of hospitalization.

**Lessons:**

Although dizziness with torsional nystagmus is known to be observed in patients with posterior canal benign paroxysmal positional vertigo, when it is sudden, persistent, and occurs independent of head repositioning, central lesions should be considered. Stroke in the brainstem should be considered first, especially in patients with vascular risk factors.

## 1. Introduction

Although dizziness/vertigo is common, identifying its cause is sometimes difficult, and critical illnesses may be hidden. Because central dizziness is sometimes associated with neurologic symptoms other than dizziness, it is important to identify other neurological symptoms via physical examination for an accurate diagnosis.^[1]^ However, in the absence of other accompanying symptoms, nystagmus is the only clue to estimate the site of the lesion. Characteristic nystagmus often predicts a responsible lesion and aids in the differential diagnosis of dizziness or vertigo.^[2]^ Although upbeat-torsional nystagmus is known to be observed in patients with posterior canal benign paroxysmal positional vertigo (BPPV), it can also be caused by central lesions. Here, we report a patient who experienced sudden onset of dizziness and characteristic spontaneous upbeat-torsional nystagmus without any other neurologic symptoms, leading to a diagnosis of pontine hemorrhage.

## 2. Case presentation

A 56-year-old woman on hemodialysis due to IgA nephropathy suddenly developed dizziness while reading before bedtime. The next morning, she visited our department with exacerbated dizziness resulting in gait disturbance. Her medical history included hypertension, hypothyroidism and dyslipidemia, but she had never previously experienced a similar episode of dizziness. She was independent in her daily life and engaged in office work before this episode. She neither drank nor smoked. On examination, she was alert, and her blood pressure was 178/106 mm Hg. Her other vital signs were normal. She did not have a headache or palpitations. Neurological examination revealed torsional nystagmus (top pole of the eyes beating to the right) with a mild upbeat component, which was not triggered by head position changes (Supplementary Video 1, Supplemental Digital Content, https://links.lww.com/MD/P4). As the dizziness and nystagmus persisted at both rest and during gazing, the patient had difficulty maintaining a standing position. There were no limitations on her extraocular movements. Dysarthria, hearing loss, tinnitus, motor paralysis, cerebellar ataxia, and sensory impairment were not observed. Although there were no signs of focal neurological deficits or accompanying symptoms, the sudden onset of dizziness and persistent and spontaneous torsional nystagmus led us to investigate central lesions as a potential cause.

**Figure 1.**
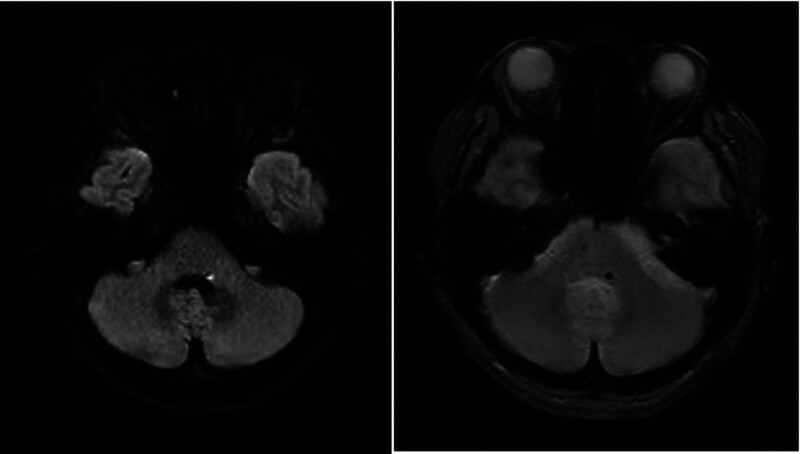
Patient brain magnetic resonance imaging. Diffusion-weighted imaging revealed a hyperintense lesion on the left dorsal pons, and a T2*-weighted image revealed a hypointense signal in the corresponding area.

Her blood cell counts and basic serum biochemistry tests were normal. The electrocardiogram was in sinus rhythm and within the normal range. In brain magnetic resonance imaging, diffusion-weighted imaging revealed a small hyperintense lesion in the left dorsal pons with a corresponding hypointense signal on T2*-weighted imaging (Fig. 1), which was consistent with pontine hemorrhage. There were no abnormalities in other areas, such as the cerebellopontine angle.

## 3. Outcomes

The patient was given antihypertensive management as a common treatment for hypertensive cerebral hemorrhage. The torsional nystagmus gradually decreased and resolved around the 4th day of hospitalization, and the dizziness was alleviated by the administration of adenosine triphosphate disodium hydrate. The patient was able to walk steadily and was discharged home on the 14th day of hospitalization.

## 4. Discussion

In a meta-analysis,^[1]^ most neurologic examination findings had low sensitivity and high specificity for differentiating central causes from peripheral etiologies in emergency department patients with acute vertigo or dizziness. Although the pooled sensitivity and specificity for spontaneous nystagmus were 52% and 42%, respectively, several specific nystagmus types, such as bidirectional, vertical, direction changing, and pure torsional nystagmus, were consistent with a central cause of dizziness/vertigo, with a pooled sensitivity of 51% and specificity of 99%. In another meta-analysis of spontaneous nystagmus in patients with acute vestibular syndrome, isolated torsional, isolated torsional-vertical and/or vertical spontaneous nystagmus were highly predictive of a central cause, with a sensitivity of 19% and specificity of 95%.^[3]^ Upbeat-torsional nystagmus is commonly observed in posterior canal BPPV, but it is usually nonpersistent and elicited after a latency of a few seconds by a change in head position, which is reproduced by the Dix–Hallpike maneuver. In a retrospective study, only 26% of posterior semicircular canal BPPV patients had spontaneous nystagmus (of which only 2% had upbeat-torsional nystagmus), which was associated with a worse outcome of dizziness/vertigo.^[4]^ Spontaneous upbeat-torsional nystagmus typically results from selective damage to the vertical semicircular canal pathways, which originate from the semicircular canal to the medial vestibular nucleus in the medulla and then cross and ascend via the medial longitudinal fasciculus (MLF) to the oculomotor and trochlear nucleus in the midbrain.^[5]^ The MLF consists of myelinated axons from the pontomedullary junction to the rostral midbrain, which provide ascending and descending connections for 3D eye movement control and is the common pathway for all conjugate adducting horizontal eye movements and the vertical–torsional vestibulo-ocular reflex. Extra-MLF lesions in the brachium conjunctivum and ventral tegmental tract for the anterior semicircular canal can also cause similar nystagmus,^[6]^ and a patient with anterior and medial medullary infarcts developed dizziness and spontaneous upbeat nystagmus, similar to our patient.^[7]^ Overall, upbeat-torsional nystagmus does not always directly suggest BPPV, and it can be observed in central lesions, especially those with sudden, persistent, and spontaneous features.

One essential component of the differential diagnosis of sudden-onset dizziness is brainstem stroke. Among patients with dizziness/vertigo due to infarctions in the vertebrobasilar circulation, 29% experienced at least one preceding isolated vertigo or dizziness episode before the onset of stroke.^[8]^ In a study of hospitalized patients who presented to the emergency department with acute isolated vertigo or dizziness, 16.8% were diagnosed with cerebral infarction, and 91% of their lesions were located in the posterior circulation.^[9]^ In an emergency room-based study comparing the acute nystagmus characteristics of posterior circulation stroke and acute vestibular neuritis, vertical-torsional nystagmus was highly specific for posterior circulation stroke, with a specificity of 98%.^[2]^ As sudden onset dizziness with spontaneous upbeat-torsional nystagmus might be a harbinger of cerebral stroke, accompanying manifestations, such as truncal ataxia and gait disturbances, should be checked; however, these assessments are sometimes difficult in patients presenting with severe dizziness and nausea. Furthermore, considering that some patients may present with only isolated vertigo or dizziness as a symptom of stroke, brain imaging studies should be performed, especially in patients with persistent and spontaneous characteristic nystagmus, such as our patient.

This case report has some limitations. Because of the single-case nature of the study, the findings may not be generalizable to all patients with vertigo. It is also important to emphasize that the absence of nystagmus in a patient with vertigo does not necessarily mean the absence of central lesions. Careful patient assessments are required, especially when the symptoms develop suddenly.

## 5. Conclusion

Although dizziness with torsional nystagmus is more commonly observed in patients with BPPV, when it is sudden, persistent, and occurs independent of head repositioning, stimulation of the MLF or extra-MLF pathways due to central lesions should be considered. Especially in patients with vascular risk factors, brainstem stroke should be considered as a differential diagnosis for sudden-onset dizziness accompanied by spontaneous and persistent upbeat-torsional nystagmus.

## Acknowledgments

The authors thank Dr Norito Kokubun, Department of Neurology, Dokkyo Medical University, for his helpful suggestion.

## Author contributions

**Conceptualization:** Mayuka Kimura.

**Data curation:** Hiroaki Fujita, Hiroki Onuma.

**Formal analysis:** Hiroki Onuma, Keisuke Suzuki.

**Writing – original draft:** Mayuka Kimura.

**Writing – review & editing:** Hiroaki Fujita, Keisuke Suzuki.

## Supplementary Material


